# O Efeito da Máscara Cirúrgica de Proteção Respiratória nos Marcadores Fisiológicos de Desempenho Aeróbio em um Corredor Recreacional

**DOI:** 10.36660/abc.20200792

**Published:** 2021-07-14

**Authors:** Danilo Marcelo Leite do Prado, Valmir Oliveira Silvino, Ewerton Gomes Vieira, Bruno Viana Rosa, Acácio Salvador Veras e Silva, Marcos Antonio Pereira dos Santos

**Affiliations:** 1 Faculdade de Medicina da Universidade de São Paulo Hospital das Clínicas São PauloSP Brasil Hospital das Clínicas da Faculdade de Medicina da Universidade de São Paulo, São Paulo, SP - Brasil; 2 Universidade Federal do Piauí Departamento de Biofísica e Fisiologia TeresinaPI Brasil Universidade Federal do Piauí - Nucleo de Estudo em Fisiologia Aplicada ao Desempenho e à Saúde (NEFADS) - Departamento de Biofísica e Fisiologia, Teresina, PI - Brasil; 3 Universidade Federal do Piauí TeresinaPI Brasil Universidade Federal do Piauí, Teresina, PI - Brasil

**Keywords:** Coronavirus-19, Pandemia, Máscara, Dispositivo de Proteção Respiratória, Atividade Física, Desempenho Aeróbio, Educação da População, Exercício, Consumo de Oxigênio, Infecção por Coronavírus

## Introdução

O advento da pandemia do Coronavírus 19 (COVID-19), que se espalhou rapidamente pelo mundo, aumentando a atenção em relação ao uso de máscara de proteção facial (MPF) não somente por profissionais da saúde, mas também pela população em geral.^1^ Neste contexto, o uso de MPF durante exercícios físicos em ambiente externo pode reduzir os riscos de infecção de COVID-19. Por outro lado, o uso da MPF pode aumentar a percepção subjetiva de dificuldade respiratória a partir da formação de microclimas dentro da máscara (ou seja, temperatura e umidade) e pela restrição do fluxo expiratório.^2^

Nos últimos anos, o número de corredores amadores aumentou significativamente entre várias populações no mundo todo, já que a corrida pode ser realizada com equipamentos mínimos e por uma ampla variedade de pessoas.^3^ É interessante notar que, durante o exercício aeróbio, a capacidade de adaptação do sistema cardiorrespiratório é de extrema importância, já que ele aumenta o transporte de oxigênio convectivo e difusivo, permitindo, assim, que o corpo atenda à demanda por oxigênio, entrega de substrato, e retirada do dióxido de carbono.^4^ Além disso, os chamados marcadores fisiológicos de desempenho aeróbio, tais como o limiar anaeróbio ventilatório, ponto de compensação respiratória, economia de corrida, e consumo máximo de oxigênio, também parecem ser importantes na definição da intensidade absoluta do exercício (ou seja, ritmo, potência).^5^

Portanto, é importante entender claramente se o uso de MPF afeta os marcadores fisiológicos do desempenho aeróbio durante a corrida. Portanto, este relato de caso avaliou o efeito do uso de MPF 1) nos marcadores fisiológicos de desempenho aeróbio e 2) na resposta cardiorrespiratória durante o exercício em um corredor recreacional.

## Relato de Caso

O voluntário que participou deste relato de caso foi um corredor saudável de 28 anos, do sexo masculino, com 10 anos de experiência em meias maratonas. Nos últimos três meses, ele correu em média 35 quilômetros por semana, com uma frequência de 3-4 sessões semanais. O participante não tinha experiência com a prática de exercícios aeróbicos com o uso de MPF. O estudo foi realizado após a obtenção do termo de consentimento informado do participante. O estudo foi aprovado pelo Comitê de Ética da Universidade Federal do Piauí, Teresina, com o número de protocolo 4,429,909.

## Exames laboratoriais

A investigação foi realizada em uma semana e consistiu em 2 fases. Na primeira fase, o voluntário realizou os testes de corrida usando uma MPF, e, na segunda fase, sem máscara (SM). Os testes foram realizados no mesmo período do dia, e com um intervalo de 48 horas entre si. O corredor foi submetido a 1) um teste de função pulmonar (TFP),^6^ 2) um teste de esforço cardiorrespiratório (TECR) para avaliar os limiares ventilatórios e o consumo máximo de oxigênio,^7^ e 3) um teste de carga retangular progressivo (PSWT) para avaliar demandas cardiorrespiratórias e economia de corrida.^8^

**Figura 1:**
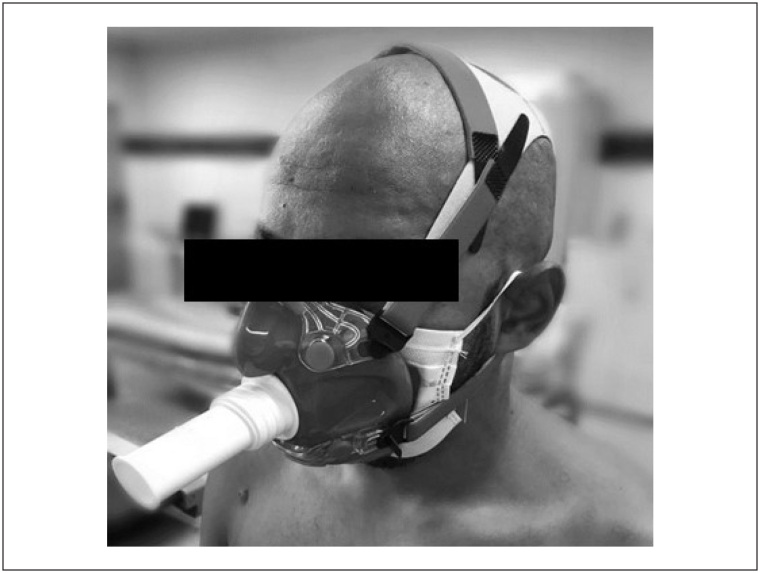
Ajuste da máscara do espirômetro sobre a máscara de proteção facial.

A máscara do espirômetro foi colocada sobre a MFP e presa por faixas na cabeça de forma a impedir vazamentos (Figura 1). O ajuste foi verificado cuidadosamente pelos investigadores e pelo voluntário para confirmar a ausência de vazamento. O ajuste correto e a prevenção de vazamentos foram confirmados antes do início de cada teste.

MPF. Neste estudo, foram utilizadas máscaras cirúrgicas fabricadas em não tecido tipo II de proteção contra a COVID-19. Sua estrutura é composta por uma camada fabricada em não tecido, material filtrante (tecido meltblown), clipe de nariz e elástico. A máscara tem formato retangular e é composta por três camadas.^9^

TFP. O do teste de função pulmonar foi realizado antes do TECR, de acordo com as recomendações da American Thoracic Society (Sociedade Torácica Americana).^10^

TECR. O teste de esforço cardiorrespiratório foi realizado em uma esteira programável (Inbramed, modelo ATL, Brasil) para determinar o consumo máximo de oxigênio (VO_2_ máx), o limiar anaeróbio ventilatório (LAV) e o ponto de compensação respiratória (PCR).^7^ A carga de exercício (velocidade) foi aumentada a cada minuto para concluir a parte incremental do teste de esforço, que durou entre 8 e 15 minutos. A velocidade inicial do teste de esforço gradual foi de 7 km/h. As análises da troca gasosa pulmonar e das variáveis ventilatórias foram feitas continuamente, respiração a respiração, durante o TECR, utilizando um sistema de análise metabólica (Ergoestik Geratherm®, Alemanha). Os seguintes critérios foram usados para definir o esforço máximo: 1) o participante demonstrou evidência subjetiva de exaustão (esforço percebido, ou seja, escala de Borg acima de 17); e 2) frequência cardíaca (FC) de pico ≥90% do máximo previsto para a faixa etária ou 3) quociente respiratório (QR) no pico do exercício ≥1,10.^11^

PSWT. 24 horas após o TECR, o corredor foi submetido a um PSWT para avaliar a economia de corrida (EC) e a resposta cardiorrespiratória em condição de ritmo estável para três domínios de exercício: 1) 80% do LAV 2) no LAV e 3) no PCR.^8^ Cada domínio de intensidade durou cinco minutos. A EC foi calculada com base na avaliação do consumo de oxigênio para uma determinada distância, utilizando a equação proposta: EC (ml O_2_.kg^−1^.km^−1^) = VO_2_ (ml.kg^−1^.h^−1^) x 60 / velocidade (Km. h^−1^).^12^ Para a avaliação da percepção subjetiva de esforço (PSE) foi utilizada a escala de Borg de 15 pontos (6 a 20), tanto no TCER quanto no PSWT.^13^

## Resultados

TFP. O corredor apresentou valores similares para os volumes pulmonares e resistência ao fluxo expiratório (Tabela 1) nas condições com MPF e SM. Entretanto, o corredor recreacional apresentou valores reduzidos de taxa de pico de fluxo expiratório (PFE) ao usar a MPF em comparação à situação SM (∆%=-25,0; Tabela 1).

**Tabela 1:** Parâmetros físicos e cardiorrespiratórios

**Medidas físicas**				
Idade (anos)		28,0		
Peso (kg)		81,0		
Altura (cm)		175,0		
**Teste de função pulmonar**		**MPF**	**SM**	**∆%**
CVF (L)		4,3	4,4	0,0
VEF_1_ (L)		4,0	4,1	0,0
VEF_1_/CVF (%)		92,3	92,3	0,0
PFE (L/s)		6,9	9,2	25,0
**Teste de esforço cardiorrespiratório**				
VO_2_max (mL.kg¹.min¹)		45,5	45,6	0,0
VO_2_max (L.min¹)		3,69	3,71	0,0
VVO_2_max (km/h)		17,0	19,0	10,5
Pico de QR (unidades)		1,21	1,18	0,02
FC de pico (bpm)		184	185	0,0
Pico de pulso de O_2_ (ml/bpm)		20,3	20,1	0,0
VE max (L.min¹)		116,2	141,1	17,6
FR (b.min¹)		57	75	24,0
VC (L.min¹)		2,1	1,9	10,0
	*Limiar anaeróbio ventilatório*			
VO_2_ (mL.kg¹.min¹)		30,5	28,5	0,07
VO_2_ (L.min¹)		2,45	2,31	0,06
Velocidade (km/h)		11,0	11,0	0,0
FC (bpm)		163	15 4	0,06
	*Ponto de compensação respiratória*			
VO_2_ (mL.kg¹.min¹)		34,9	32,7	0,06
VO_2_ (L.min¹)		2,82	2,65	0,06
Velocidade (km/h)		13,0	13,0	0,0
FC (bpm)		17 4	16 5	0,05

Símbolos e abreviaturas: MPF: máscara de proteção facial; SM: sem máscara; CVF: capacidade vital funcional; VEF_1_: volume expiratório forçado em 1 segundo; VEF_1_/CVF: relação volume expiratório forçado em 1 segundo e capacidade vital funcional; PFE: pico de fluxo expiratório; VO_2_max: consumo máximo de oxigênio; VVO_2_max: velocidade de captação máxima de oxigênio; QR: quociente respiratório; FC: frequência cardíaca; VE: ventilação pulmonar; FR: frequência respiratória; VC: volume corrente; L: litros; L/s: litros por segundos; km/h: quilômetros por hora; bpm: batimentos por minuto.

TECR. Para ambas as condições, os dados deste estudo apresentaram valores semelhantes de VO_2_ máx, FC de pico, e pulso de O_2_. Entretanto, o corredor recreacional apresentou VVO_2_ máx, ventilação pulmonar (VE), e frequência respiratória (FR) menores enquanto utilizava a MPF (∆%=-10,5, -17,6 e -24.0, respectivamente; Tabela 1). Por outro lado, os resultados deste estudo apontaram valores de volume corrente (VC) mais altos com o uso da máscara (∆%=10,0, Tabela 1).

Em relação aos limiares ventilatórios, o voluntário apresentou valores de velocidade similares para ambas as condições. Entretanto, nossos achados apresentaram diferenças no VO_2_ (mL.kg¹.min¹ e L.min¹) e na FC (Tabela 1).

**Figura 2:**
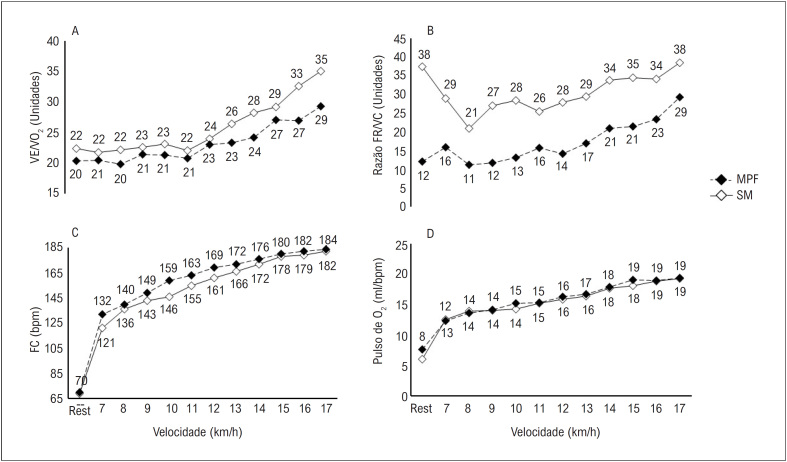
Resposta cardiorrespiratória durante o TECR em um corredor recreacional com e sem o uso de MPF. Painel A= VE/VO_2_; Painel B= razão FR/VC; Painel C= FC; Painel D= pulso de O_2_. MPF: máscara de proteção facial; SM: sem máscara; TECR: teste de esforço cardiorrespiratório; VE/V0_2_: equivalente ventilatório para oxigênio; razão FR/VC: razão da frequência respiratória e volume corrente; FC: frequência cardíaca.

A resposta cardiorrespiratória durante o TECR está descrita na Figura 2. Em relação ao VE/VO_2_, o corredor apresentou valores menores enquanto usava a MPF, em comparação à situação SM (Figura 2A). Da mesma forma, foi observada uma redução na razão FR/VC (Figura 2B). Em contrapartida, o voluntário demonstrou uma resposta de FC mais alta enquanto usava a MPF, em comparação à situação SM (Figura 2C). Além disso, observou-se uma resposta semelhante de pulso de O_2_ para ambas as condições (Figura 2D).

**Figura 3:**
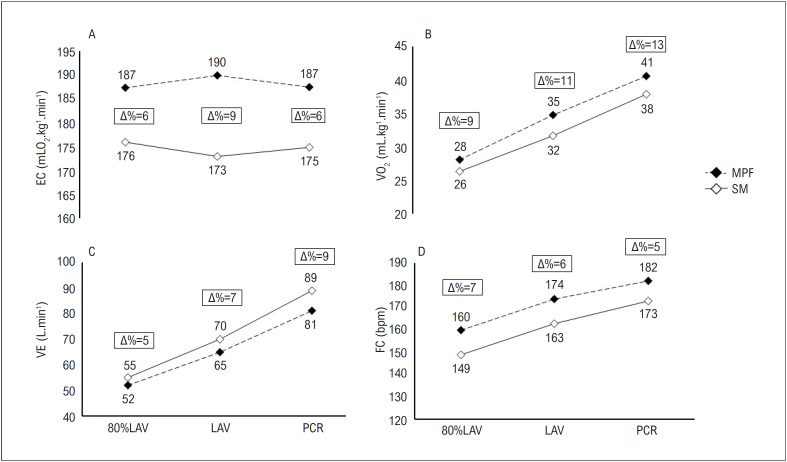
Resposta cardiorrespiratória durante o PSWT em um corredor recreacional com e sem o uso de MPF. Painel A= EC; Painel B= VO_2_; Painel C= VE; Painel D= FC. MPF: máscara de proteção facial; SM: sem máscara; PSWT: teste de carga retangular progressivo; EC: economia de corrida; VE: ventilação pulmonar; FC: frequência cardíaca; LAV: limiar anaeróbio ventilatório; PCR: ponto de compensação respiratório.

PSWT. O corredor recreacional apresentou valores mais altos de EC, VO_2_ e FC enquanto usava a MPF (Figuras 3A, B e D, respectivamente). Por outro lado, o voluntário demonstrou valores de VE menores enquanto usava a MPF, em comparação à situação SM (Figura 3C).

**Figura 4:**
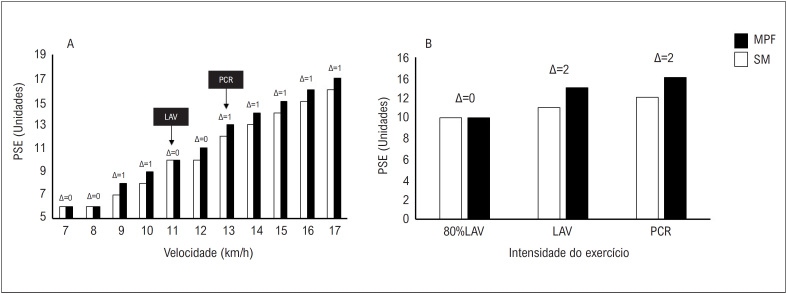
Classificação da percepção subjetiva do esforço durante o TECR (painel A) e PSWT (painel B) em um corredor recreacional com e sem o uso de MPF. MPF: máscara de proteção facial; SM: sem máscaras; TECR: teste de esforço cardiorrespiratório; PSWT: teste de carga retangular progressivo; PSE: percepção subjetiva de esforço; LAV: limiar anaeróbio ventilatório; PCR: ponto de compensação respiratória.

PSE. Os resultados deste estudo demonstraram que a PSE durante o TECR foi mais alta com o uso da MPF, quando comparado à condição controle (∆=1 ponto; nas velocidades = 9,10,13,14,15,16, e 17 km/h; Figura 4A). Da mesma forma, durante o PSWT, o participante apresentou níveis elevados de PSE enquanto usava a MPF no LAV (∆=2 pontos) e no PCR (∆=2 pontos).

## Discussão

Os dados deste estudo sugerem que o uso de máscara de proteção facial afetou a tolerância ao exercício e economia de corrida em um corredor recreacional. Consta na literatura que capacidade cardiopulmonar de exercício e sensação de conforto são reduzidos pelo uso de máscaras cirúrgicas, e são significativamente reduzidos com o uso de máscaras faciais FFP2/N95 em sujeitos saudáveis.^14^ Além disso, observou-se que o uso de máscara cirúrgica não afeta a capacidade de função cardiopulmonar durante exercícios que envolvem pedalada.^15^ Entretanto, até onde se sabe, este é o primeiro relato de caso que avaliou especificamente o efeito de uma máscara de proteção facial nos marcadores fisiológicos de desempenho aeróbio em um corredor recreacional.

É interessante notar que uma corrida de intensidade auto selecionável depende dos marcadores psicofisiológicos de desempenho aeróbio.^5^^,^^16^ Os resultados do presente estudo apresentaram uma resposta similar em relação ao VO_2_ máx e aos limiares ventilatórios com o uso de máscara. Por outro lado, o corredor recreacional apresentou menor velocidade na intensidade do VO_2_ max com o uso da MPF. É importante notar que os achados deste estudo sugerem que, embora a capacidade de transporte e utilização de oxigênio tivesse sido preservada, o corredor apresentou menor tolerância ao exercício. Vale ressaltar que o participante também apresentou redução da EC com o uso da MPF, o que sugere maiores demandas de oxigênio durante a corrida em comparação com a condição SM.

Outro ponto interessante é como a resposta ventilatória se adapta ao uso da MPF durante o TECR e o PSWT. Durante o exercício físico, há um aumento da taxa metabólica e, consequentemente, em demandas ventilatórias. Vale notar também que o corredor demonstrou resposta ventilatória menor durante o exercício com o uso da MPF. Especificamente, os resultados deste estudo apresentaram valores menores da razão VE/VO_2_, sugerindo maior eficiência ventilatória com o uso de MPF. Entretanto, apesar da melhoria da eficiência ventilatória, o voluntário apresentou maior desconforto respiratório com o uso da MPF.

Com base nos achados mencionados acima, surge a seguinte pergunta: quais são os mecanismos fisiológicos subjacentes ao desconforto respiratório com o uso da MPF? De fato, sugere-se que fatores associados ao aumento da obstrução do fluxo expiratório podem estar relacionados. Neste contexto, os resultados deste estudo apresentaram níveis menores de TPFE e VE no pico do exercício. Além disso, em relação ao padrão respiratório, o corredor apresentou uma razão FR/VC reduzida com o uso da MPF. É importante observar que a razão FR/VC é utilizada para avaliar indiretamente as interações mecânicas/ventilatórias durante o exercício.^17^ Neste sentido, para um determinado resultado ventilatório, o corredor aumentou o volume corrente de forma mais aguda que a frequência respiratória, aumentando consequentemente o esforço da musculatora inspiratória e, portanto, a sensação de esforço respiratório.

Por último, os dados deste estudo sugerem uma associação entre os esforços da musculatura inspiratória e um aumento das demandas de oxigênio e da resposta de frequência cardíaca durante o exercício com o uso de MPF. Neste contexto, Harms et al.^18^ demonstraram que a descarga da musculatura inspiratória durante o exercício aeróbico está associada com redução do VO_2_ e nível de dispnéia. Por exemplo, há evidências de que um esforço inspiratório maior durante o exercício está relacionado ao aumento da ativação do metaborreflexo do músculo inspiratório e, portanto, do fluxo simpático.^19^ É interessante notar que, no mesmo estudo,^19^ os autores observaram que cinco semanas de treinamento da musculatura inspiratória foram suficientes para aumentar a sua resistência e atenuar o aumento da frequência cardíaca durante o exercício.

## Aplicações práticas

O presente relato de caso indica que a tolerância ao exercício e a economia de corrida foram reduzidas quando o corredor recreacional usou uma máscara de proteção facial. Além disso, os achados deste estudo sugerem uma possível associação entre aumento da obstrução do fluxo expiratório, maior sobrecarga mecânica na musculatura inspiratória demandas cardiovasculares mais altas durante o exercício aeróbio. É importante destacar que cada teste durou menos de 20 minutos, o que ajudou a manter a condição e o funcionamento da máscara.

Portanto, com base nos achados do presente relato de caso, sugere-se que as seguintes estratégias sejam usadas para minimizar o desconforto respiratório durante o exercício aeróbio com o uso de MPF: 1) inclusão do treinamento da musculatura inspiratória no programa de treinamento aeróbio; 2) prescrição da intensidade do exercício aeróbio com base na frequência cardíaca de reserva (FCR) (ou seja, método Karvonen) ou limiares ventilatórios (ou seja, LAV e PCR); 3) prescrição da intensidade de exercícios aeróbios em três zonas, ou seja Zona 1 - fácil (<LAV), Zona 2 - moderada (entre LAV e PCR); e Zona 3 - alto impacto (>PCR); e 4) para indivíduos sedentários e pacientes com doenças crônicas, sugere-se que, nos estágios iniciais do programa de treinamento aeróbio, o exercício seja de baixa intensidade (ou seja, <LAV ou 30-40% da RFC).

## Conclusões

Os resultados deste estudo sugerem que o corredor recreacional, ao usar a MPF, apresentou: 1) diminuição da resistência ao exercício apesar da resposta semelhante no VO_2_ máx e limiares ventilatórios; 2) diminuição economia de corrida; 3) um aumento na demanda cardiovascular em relação à resposta da frequência cardíaca; 4) aumento da carga sobre os músculos respiratórios com o padrão respiratório adotado durante o exercício, apesar da menor demanda ventilatória; e 5) um aumento na classificação da percepção subjetiva do esforço e no desconforto respiratório.
