# Supplementary material for: O Efeito da Máscara Cirúrgica de Proteção Respiratória nos Marcadores Fisiológicos de Desempenho Aeróbio em um Corredor Recreacional
Source: Arq Bras Cardiol. 2021 Jul 14;117(1 Supl 1):23–8. [Article in Portuguese] doi: 10.36660/abc.20200792 (PMC8291894; doi:10.36660/abc.20200792)
Supplement: Translation

# Translation: The Effect of Respiratory Protective Surgical Mask on Physiological Markers of Endurance Performance in a Recreational Runner

**Authors:** Danilo Marcelo Leite do Prado, Valmir Oliveira Silvino, Ewerton Gomes Vieira, Bruno Viana Rosa, Acácio Salvador Veras e Silva, Marcos Antonio Pereira dos Santos

**Affiliations:** 1 São PauloSP Brazil Hospital das Clínicas da Faculdade de Medicina da Universidade de São Paulo, São Paulo, SP - Brazil; 2 TeresinaPI Brazil Universidade Federal do Piauí - Nucleo de Estudo em Fisiologia Aplicada ao Desempenho e à Saúde (NEFADS) - Departamento de Biofísica e Fisiologia, Teresina, PI - Brazil; 3 TeresinaPI Brazil Universidade Federal do Piauí, Teresina, PI - Brazil

**Keywords:** Coronavirus-19, Pandemics, Facial Mask, Respiratory Protective Devices, Resistance Performance, Physical Activity, Population Education, Exercise, Oxygen Consumption, Coronavirus-19 Infection

## Introduction

The advent of the Coronavirus 19 (COVID-19) pandemic, which has quickly spread worldwide, has raised the attention regarding the use of respiratory protective face masks (PFM) not only by healthcare personnel, but also the general population.^1^ In this context, wearing a PFM during physical exercise in an external environment can reduce COVID-19 infection risks. On the other hand, the use of PFM can increase the subjective perception of breathing difficulty through the formation of microclimates inside the face mask (i.e., temperature and humidity) and airflow restriction.^2^

In recent years, the number of amateur runners has significantly increased among many populations around the world, as running can be performed with minimal equipment, and by a broad variety of people.^3^ Interestingly, during endurance exercise, the adaptability of the cardiorespiratory system is of paramount importance, as it increases both convective and diffusive oxygen transport, thus enabling the body to meet the demands for oxygen, substrate delivery, and carbon dioxide removal.^4^ Moreover, the so-called physiological markers of endurance performance, such as ventilatory anaerobic threshold, respiratory compensation point, running economy, and maximal oxygen uptake, also seem to be important in determining absolute exercise intensity (i.e., pace, power output).^5^

In this light, it is important to have a clear understanding of whether or not the use of a PFM affects physiological markers of endurance performance during running. Therefore, our case-study evaluated the effect of wearing a PFM on 1) physiological markers of endurance performance and 2) cardiorespiratory response during exercise in a recreational runner.

## Case Report

The volunteer who participated in this case study was a healthy 28-year-old male runner with 10 years of half-marathon running experience. In the last three months, he ran an average of 35 kilometers per week with a frequency of 3-4 weekly sessions. The participant had no experience with the practice of aerobic exercise while wearing a protective face mask. The study was carried out after informed consent from the participant. The study was approved by the Research Ethics Committee of the Federal University of Piauí, Teresina, Brazil under protocol number 4.429.909.

## Laboratory Assessment

This investigation was carried out in one week and consisted of 2 phases. In the first phase, the volunteer performed the running tests while wearing a PFM and no mask (NM) in the second phase. The tests were performed at the same time of day, and with an interval of at least 48 hours between the tests. The runner underwent 1) a pulmonary function test (PFT),^6^ 2) a cardiorespiratory exercise test (CPET) to assess ventilatory thresholds and maximal oxygen consumption,^7^ and 3) a progressive square-wave test (PSWT) to evaluate both cardiorespiratory demands and running economy.^8^

The spirometer mask was placed over the PFM and fixed with head straps in a leak-proof manner (Figure 1). The fitting was thoroughly checked for the absence of leakage by the investigators and the volunteer. The correct fitting and leak tightness were confirmed before each test was started.

**Figure 1:** Fitting of the spirometer mask to the protective face mask.

PFM. In this study, a disposable non-woven COVID-19 type II surgical mask was used. Its structure comprises a non-woven fabric layer, filter material (melt-blown fabric), nose clip, and mask belt. The mask is rectangular in shape and contains three layers.^9^

PFT. The pulmonary function test measurement was carried out before the CPET, according to American Thoracic Society recommendations.^10^

CPET. The cardiorespiratory exercise test was conducted using a programmable treadmill (Inbramed model ATL, Brazil) in order to determine maximal oxygen consumption (VO_2_ max), ventilatory anaerobic threshold (VAT), and the respiratory compensation point (RCP).^7^ The exercise workload (speed) was increased every one minute to complete the incremental part of the exercise test, which lasted between 8 and 15 minutes. The starting speed in the graded exercise test was 7 km/h. Gas exchange and ventilatory variables were measured continuously breath-by-breath during the gas exchange test, using a metabolic analyzer system (Ergoestik Geratherm®, Germany). The following criteria were used to define maximal effort: 1) participant demonstrated subjective evidence of exhaustion (perceived exertion, i.e., Borg scale above 17); and either 2) peak heart rate (HR) ≥90% age-predicted maximum or 3) maximal respiratory exchange ratio (RER) ≥1.10.^11^

PSWT. 24 hours after the CPET, the runner underwent a PSWT to determine both the running economy (RE) and cardiorespiratory response in steady-state condition at three exercise domains: 1) at 80% VAT, 2) at VAT, and 3) at RCP.^8^ Each intensity domain lasted five minutes. The RE was calculated in terms of oxygen cost to cover a given distance using the proposed equation: RE (ml O_2_.kg^−1^.km^−1^) = VO_2_ (ml.kg^−1^.h^−1^) x 60 / speed (Km. h^−1^).^12^ The rating of perceived exertion (RPE) was used in both CPET and PSWT with the 15-point (6-20) Borg scale.^13^

## Results

PFT. The runner showed similar values for lung volumes and airflow resistance (Table 1) in both PFM and NM conditions. However, the recreational runner demonstrated lower values of peak expiratory flow rate (PEFR) while wearing the PFM when compared to NM (∆%=-25.0; Table 1).

**Table 1:** Physical and Cardiorespiratory parameters

**Physical measurements**				
Age (years)		28.0		
Weight (kg)		81.0		
Height (cm)		175.0		
**Pulmonary Function Test**		**PFM**	**NM**	**∆%**
FVC (L)		4.3	4.4	**0.0**
FEV_1_ (L)		4.0	4.1	**0.0**
FEV_1_ /FVC (%)		92.3	92.3	**0.0**
PEFR (L/s)		6.9	9.2	**25.0**
**Cardiorespiratory Exercise Test**				
VO_2_ max (mL.kg¹.min¹)		45.5	45.6	**0.0**
VO_2_ max (L.min¹)		3.69	3.71	**0.0**
VVO_2_ max (km/h)		17.0	19.0	**10.5**
Peak RER (Units)		1.21	1.18	**0.02**
Peak HR (bpm)		184	185	**0.0**
Peak O_2_ pulse (ml/bpm)		20.3	20.1	**0.0**
VE max (L.min¹)		116.2	141.1	**17.6**
RR (b.min¹)		57	75	**24.0**
TV (L.min¹)		2.1	1.9	**10.0**
	*Ventilatory anaerobic threshold*			
VO_2_ (mL.kg¹.min¹)		30.5	28.5	**0.07**
VO_2_ (L.min¹)		2.45	2.31	**0.06**
Speed (km/h)		11.0	11.0	**0.0**
HR (bpm)		163	154	**0.06**
	*Respiratory compensation point*			
VO_2_ (mL.kg¹.min¹)		34.9	32.7	**0.06**
VO_2_ (L.min¹)		2.82	2.65	**0.06**
Speed (km/h)		13.0	13.0	**0.0**
HR (bpm)		174	165	**0.05**

Symbols and abbreviations: PFM: protective face mask; NM: no mask; FVC: functional vital capacity; FEV_1_: forced expiratory volume in 1 second; FEV_1_/FVC: forced expiratory volume in 1 second to functional vital capacity ratio; PEFR: peak expiratory flow rate; VO_2_ max: maximal oxygen uptake; VVO_2_: the speed at maximal oxygen uptake; RER: respiratory exchange ratio; HR: heart rate; VE: pulmonary ventilation; RR: respiratory rate; TV: tidal volume; L: liters; L/s: liters per seconds; km/h: kilometers per hour; bpm: beats per minute.

CPET. For both conditions, our data showed similar values for VO_2_ max, peak HR, and O_2_ pulse. However, the recreational runner presented lower VVO_2_ max, pulmonary ventilation (VE), and respiratory rate (RR) while wearing the PFM (∆%=-10.5, -17.6, and -24.0, respectively; Table 1). On the other hand, our results showed higher volume tidal (VT) values with face mask use (∆%=-10.0, Table 1).

Regarding ventilatory thresholds, the volunteer demonstrated similar speed values for both conditions. However, our results showed differences in VO_2_ (mL.kg¹.min¹and L.min¹) and HR values (Table 1).

The cardiorespiratory response during CPET is shown in Figure 2. With respect to VE/VO_2_, the runner demonstrated lower values while wearing PFM when compared to NM. (Figure 2A). A similar finding was observed for the RR/VT ratio (Figure 2B). By contrast, the volunteer demonstrated higher HR response while wearing the PFM compared to NM (figure 2C). Moreover, a similar response was observed in O_2_ pulse for both conditions (Figure 2D).

**Figure 2:** Cardiorespiratory response during CPET in a recreational runner with and without PFM use. Panel A= VE/VO_2_; Panel B= RR/VT ratio; Panel C= HR; Panel D= O_2_ pulse. PFM: protective face mask; NM: no mask; CPET: cardiorespiratory exercise test; VE/VO_2_: ventilatory equivalent for oxygen; RR/VT ratio: respiratory rate to volume tidal ratio; HR: heart rate.

PSWT. The recreational runner showed greater values for RE, VO_2,_ and HR while wearing the PFM (Figures 3A, B, and D, respectively). However, our data demonstrated lower values of VE while wearing the PFM compared to NM (Figure 3C).

**Figure 3:** Cardiorespiratory response during PSWT in a recreational runner with and without PFM use. Panel A= RE; Panel B= VO_2_; Panel C= VE; Panel D= HR. PFM: protective face mask; NM: no mask; PSWT: progressive square wave test; RE: running economy; VE: pulmonary ventilation; HR: heart rate.

RPE. Our results showed that RPE during the CEPT was greater while wearing the PFM when compared to the control condition (∆=1 point; at speeds= 9,10,13,14,15,16, and 17 km/h; Figure 4A). Likewise, during PSWT, the participant showed higher RPE levels while wearing the PFM for both VAT (∆=2 points) and RCP (∆=2 points).

**Figure 4:** Rating of perceived exertion during CPET (panel A) and PSWT (panel B) in a recreational runner with and without PFM use. PFM: protective face mask; NM: no mask; RPE: rating of perceived exertion; VAT: ventilatory anaerobic threshold; RCP: respiratory compensation point.

## Discussion

Our data suggest that the use of a protective face mask affected the exercise tolerance and running economy in a recreational runner. It has already been reported that both cardiopulmonary exercise capacity and comfort are reduced by surgical masks and highly impaired by FFP2/N95 face masks in healthy subjects.^14^ Moreover, it has been observed that wearing a surgical mask does not affect cardiopulmonary function capacity during pedaling exercise.^15^ However, to the best of our knowledge, this is the first case study to specifically evaluate the effect of a protective face mask on physiological markers of endurance performance in a recreational runner.

Interestingly, a self-paced running intensity is dependent on both psychological and physiological markers of endurance exercise.^5^^,^^16^ In the present case study, our results showed a similar response to both VO_2_ max and ventilatory thresholds when wearing a face mask. On the other hand, the recreational runner showed lower speed at VO_2_ max while wearing the PFM. Importantly, our findings suggest that, although the ability of oxygen transport and use is preserved, the runner presented lower exercise tolerance. It is important to note that the participant also demonstrated a worsening in RE while wearing PFM, which suggests greater oxygen demands during running when compared to the NM condition.

Another interesting point is how the ventilatory response adapts to the use of a protective face mask during CPET and PSWT. During physical exercise, there is an increase in metabolic rate and, consequently, in ventilatory demands. It is also worth noting that the runner demonstrated lower ventilatory response during exercise with the use of PFM. More specifically, our results demonstrated lower values for the VE/VO_2_ ratio, suggesting greater ventilatory efficiency with PFM use. However, despite the improvement in the ventilatory efficiency, the volunteer showed greater respiratory discomfort wearing PFM.

Based on the above findings, the following question emerges: what physiological mechanisms underlie respiratory discomfort with wearing PFM? In fact, we suggest that factors associated with an increase in airflow impedance may be related. In this context, our results demonstrated lower levels of PEFR and VE at the peak of the exercise. Furthermore, regarding breathing patterns, the runner showed a lower RR/VT ratio when wearing a face mask. Importantly, the RR/VT ratio is used to indirectly evaluate mechanical/ventilatory interactions during exercise.^17^ In this sense, for a given ventilatory output, the runner increased the tidal volume more sharply than the respiratory rate, consequently increasing the inspiratory muscle effort and, therefore, the sense of respiratory effort.

Finally, our data suggested an association between inspiratory muscle effort and increased both oxygen demands and heart rate response during exercise with face mask use. In this context, Harms et al.^18^ demonstrated that inspiratory muscle unloading during aerobic exercise was associated with reduced VO_2_ and dyspnea ratings. For instance, there is evidence that greater inspiratory effort during exercise is related to increased activation inspiratory muscle metaboreflex and, thus, sympathetic outflow.^19^ Notably, in the same study,^19^ the authors observed that five weeks of inspiratory muscle training was capable of increasing inspiratory muscle strength and attenuating the rise in heart rate during exercise.

## Practical Applications

The present case study indicates that both exercise tolerance and running economy are worsened when the recreational runner wore a protective face mask. Additionally, our findings suggest a possible association between increased airflow impedance, greater inspiratory muscle mechanical overload, and higher cardiovascular demands during endurance exercise. It is important to point out that each test lasted less than 20 minutes, which helped maintain the condition and functioning of the mask.

Thus, based on the findings of the present case study, we suggest the following strategies to minimize respiratory discomfort during aerobic exercise when wearing a PFM: 1) inspiratory muscle training inclusion in the endurance training program; 2) prescription of aerobic exercise intensity based on percentages of heart rate reserve (HRR) (i.e., Karvonen method) or ventilatory thresholds (i.e., VAT and RCP); 3) prescription of the aerobic exercise intensity into three zones, i.e., Zone 1 - easy (<VAT); Zone 2 - moderate (between VAT and RCP); and Zone 3 - high intensity (> RCP); and 4) For both sedentary individuals and patients with chronic diseases, we suggest that, in the early stages of the endurance training program, the aerobic exercise may be of low intensity (i.e., < VAT or 30- 40% HRR).

## Conclusions

In conclusion, our results suggest that the recreational runner, while wearing a PFM, showed: first, decreased exercise tolerance despite similar response to both VO_2_ max and ventilatory thresholds; second, a worsening of the running economy; third, an increase in cardiovascular demand regarding heart rate response; fourth, despite the lower ventilatory demand, the breathing pattern adopted during exercise increased the burden on the respiratory muscles; and last, an increase in rating of perceived exertion and respiratory discomfort.
